# In Vitro and In Vivo Biological Assessments of 3D-Bioprinted Scaffolds for Dental Applications

**DOI:** 10.3390/ijms241612881

**Published:** 2023-08-17

**Authors:** Nurulhuda Mohd, Masfueh Razali, Mh Busra Fauzi, Noor Hayaty Abu Kasim

**Affiliations:** 1 Department of Restorative Dentistry, Faculty of Dentistry, Universiti Kebangsaan Malaysia, Kuala Lumpur 50300, Malaysia; nurulhuda.mohd@ukm.edu.my; 2 Centre for Tissue Engineering and Regenerative Medicine, Faculty of Medicine, Universiti Kebangsaan Malaysia, Kuala Lumpur 56000, Malaysia; fauzibusra@ukm.edu.my; 3 Department of Restorative Dentistry, Faculty of Dentistry, Universiti Malaya, Kuala Lumpur 50603, Malaysia; 4 Dean Office, Faculty of Dentistry, Universiti Kebangsaan Malaysia, Kuala Lumpur 50300, Malaysia

**Keywords:** regenerative dentistry, dental tissues regeneration, three-dimensional bioprinting, cell laden, bioink, tissue engineering

## Abstract

Three-dimensional (3D) bioprinting is a unique combination of technological advances in 3D printing and tissue engineering. It has emerged as a promising approach to address the dilemma in current dental treatments faced by clinicians in order to repair or replace injured and diseased tissues. The exploration of 3D bioprinting technology provides high reproducibility and precise control of the bioink containing the desired cells and biomaterial over the architectural and dimensional features of the scaffolds in fabricating functional tissue constructs that are specific to the patient treatment need. In recent years, the dental applications of different 3D bioprinting techniques, types of novel bioinks, and the types of cells used have been extensively explored. Most of the findings noted significant challenges compared to the non-biological 3D printing approach in constructing the bioscaffolds that mimic native tissues. Hence, this review focuses solely on the implementation of 3D bioprinting techniques and strategies based on cell-laden bioinks. It discusses the in vitro applications of 3D-bioprinted scaffolds on cell viabilities, cell functionalities, differentiation ability, and expression of the markers as well as the in vivo evaluations of the implanted bioscaffolds on the animal models for bone, periodontal, dentin, and pulp tissue regeneration. Finally, it outlines some perspectives for future developments in dental applications.

## 1. Introduction

Three-dimensional (3D) bioprinting has been shown to be a promising technology in addressing current challenges in the field of regenerative medicine. The availability of a broad choice of suitable bioinks has enabled rapid and precise fabrication of stable constructs to restore, maintain, and enhance lost or injured tissues [1,2]. In medical applications, certain 3D-bioprinted tissues such as skin and blood vessels have been successfully engineered in the laboratory. These tissues have been implanted in patients in a limited number of clinical trials [3]. Even though 3D bioprinting has encouraging prospects in medical therapy in general, research on specific applications in dentistry is believed to still be at the preliminary stage. This technology, in combination with advanced tissue engineering, has great potential to tackle some of the key challenges in craniofacial reconstruction and the functional regeneration of dental tissues such as the alveolar bone, periodontal ligament, and the dentin–pulp complex [4].

**Figure 1:**
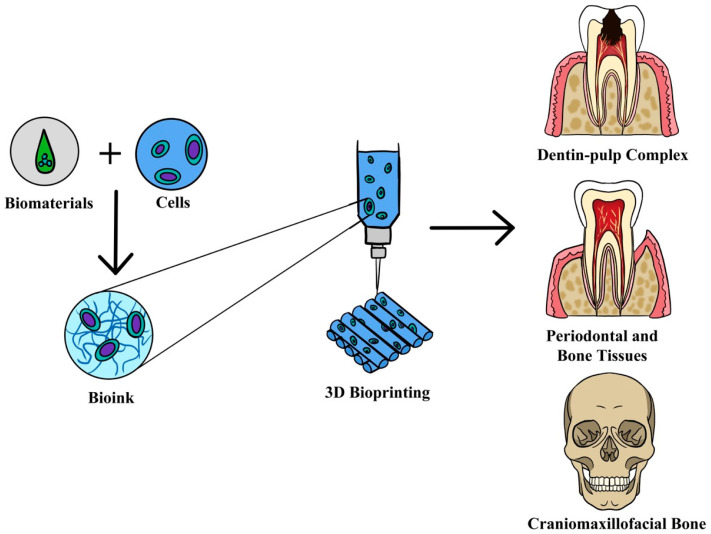
Three-dimensional bioprinted construct for dental tissue engineering applications. Reproduced with permission from Mohd et al. (2022) [4] (http://creativecommons.org/licenses/by/4.0/) (accessed on 12 June 2023).

Craniofacial defects resulting from trauma, tumor, congenital anomalies, or infection present challenging reconstructive procedures. Precise placement of multiple tissues is needed to recapitulate the complex geometries of tissue anatomy and function [5,6]. This is also true for bone regeneration on severely resorbed alveolar bones prior to implant placement when replacing missing teeth [7]. Another clinical challenge is to treat periodontitis, the sixth most prevalent disease worldwide, when current therapies cannot repair the destroyed alveolar bone and restore the functionality of the periodontally involved teeth [8,9]. In order to achieve successful periodontal regeneration, the formation of new cementum, a functionally oriented periodontal ligament, and the restoration of alveolar bone height need to be established simultaneously. Root canal treatment is unable to achieve dental pulp regeneration because the therapy sacrifices the dental pulp tissue and fills it with inert biomaterial. Even though the treatment success rate is high, the biological functions of the tooth cannot be re-established, namely sensory stimulation, dentin formation, and immune response against microorganisms [10]. Therefore, there is a need for the revascularization or regeneration of dental pulp, especially in immature permanent teeth for the maintenance of function. These clinical challenges and drawbacks of conventional treatments in dentistry have led to the exploration of advanced 3D bioprinting technology. Figure 1 shows 3D bioprinting in dental applications. This new approach enables the fabrication of personalized scaffolds to a patient’s specific tissue constructs with complex architectures. It involves layer-by-layer precise deposition of cells, DNA, growth factors, and other bioactive components [11,12]. This is in contrast to the 3D printing technology or additive manufacturing which involve fabricating biomaterial scaffolds using fused deposition modeling (FDM), digital light processing (DLP), selective laser sintering (SLS), or stereolithography (SLA) without cell incorporation during the printing process [13,14]. In the dental field, 3D printing is able to create successive layers of implantable materials from natural and synthetic polymers, metals, ceramics, or composites for craniofacial and dental implants, as well as scaffolds for tissue regeneration [7,15,16,17]. There are essential steps required after printing in order to achieve the appropriate surface finishing, accuracy of design, and material properties of the final 3D patient-specific anatomical scaffolds [18]. Since the cells are not a component in the printed structures, the biocompatibility of the 3D scaffolds with cells, tissues, and the humoral system is paramount to prevent any adverse responses [19,20].

At present, 3D bioprinting technology which incorporates cells during printing has become the preferred choice compared to the conventional method of cell seeding. The conventional tissue engineering strategy involves seeding the cells onto scaffolds resulting in cell proliferation and differentiation into functioning tissues [21]. The limitation of this conventional approach is potentially poor cellular performance and cell loss due to ineffective cell seeding, penetration and migration [21,22]. The conventional method of seeding the cells has been shown to encounter difficulty in fabricating the vascular system in the scaffold and simultaneously constructing tissues that are thicker and complex [21]. Therefore, the emerging technology of 3D bioprinting could resolve the limitations of the traditional regenerative approach.

**Figure 2:**
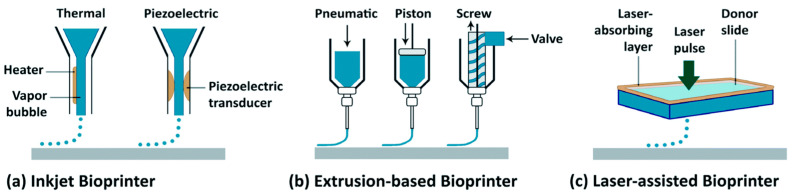
Common three-dimensional bioprinting techniques. Reproduced with permission from Gungor-Ozkerim et al. (2018) [36]; permission conveyed through Copyright Clearance Center, Inc.

The 3D bioprinting technique that is widely used in dental applications is extrusion-based, followed by inkjet and laser assisted bioprinting [4] as illustrated in Figure 2. Extrusion-based bioprinting uses either a pneumatic or a piston/screw-based system to eject a continuous stream of bioink. The pneumatic system uses compressed air whereas the mechanical type utilizes a screw or piston to force the bioink out of the nozzle. The extrusion-based method is a preferred method for fabricating 3D constructs because of the wide selection of biomaterials with high concentrations and viscosities [23]. The appropriate viscosity of the biomaterials using the extrusion technique is in the range of 30–60 × 10^7^ mPa/s [24]. Accurate printing, fast speed and low cost are among the advantages of this approach. It can also fabricate scaffolds with intricate structures and pores with high cell density [25]. However, one of the limitations of extrusion technique is the difficulty in achieving adequate mechanical stability and structural integrity of large free-form structures [26,27]. In addition, dispensing pressure and shear stress generated by the system could also affect cell viability [28]. The selection of biomaterials based on their viscosities is crucial to prevent nozzle clogging during extrusion [29]. The inkjet-based technique is another type of the nozzle-based 3D bioprinting method which could either be thermal or acoustic [30]. The thermal inkjet has a heating system installed in the printer head that creates bubbles ejecting the bioink into droplets. The acoustic inkjet employs piezoelectricity to create pulses in the printer head and produce droplets out of the nozzle. High processing speed and high resolution with low cost, ease of operation and capability for tweaks and modification makes the inkjet one of the preferred bioprinting approaches. This technique requires lower biomaterial viscosities in the range of 3.5–12 mPa/s compared to the extrusion-based technique because the inkjet produces droplets of the material rather than filaments [24]. However, this technique is limited by cell concentrations of <5 × 10^6^ cells/mL and the possibility of nozzle clogging. The other drawback is a lack of effective structural integrity because of the low-viscosity biomaterials [31]. Laser-assisted bioprinting (LAB), on the other hand, uses the principle of laser-induced forward transfer (LIFT). This approach uses a high-energy laser pulse to create high-pressure bubbles in the biomaterial layer to eject suspended bioink directly to a predetermined place. LAB is a nozzle-free and non-contact printing method which eliminates the challenges in nozzle-based 3D bioprinting systems [32]. This technique involves high precision of the construct up to a nano-scale size with high resolution printing, which allows the printing out of high cell densities (1 × 10^8^ cells/mL) [33]. However, the disadvantage of using LAB is the time-consuming factor caused by its slow printing speed which in some situations is not suitable if rapid fabrication is required due to dehydration issues [34,35]. Furthermore, the slow printing speed also makes it difficult to fabricate a larger construct and handle heterogeneous cells [34]. The stress induced by the ultraviolet (UV) light from the laser, for example, may introduce the possibility of tissue damage [24].

Bioink is an important element in 3D bioprinting. It is a suspension of cells that may contain biomaterials and biologically active components [37]. An ideal bioink should be highly printable into stable 3D structures while providing an appropriate environment for encapsulated cells during and after printing for long-term tissue formation. In dental applications, the materials in the bioink are derived from natural polymers such as collagen (col), gelatin (gel), alginate, hyaluronic acid (HA), chitosan, cellulose, glycerol, fibrin or synthetic polymers like poly(ethylene glycol) dimethacrylate (PEGDA) and synthetic copolymer of poly(ethylene glycol) and poly(propylene glycol) (Poloxamer-407). The modified naturally derived polymers such as gelatin methacryloyl (GelMA) and methacrylated hyaluronic acid (MeHA) have also been used for dental applications. The bioinks were also used in combinations with bioceramic materials such as hydroxyapatite (HAp), tricalcium phosphate (TCP) and nanosilicates to achieve stable constructs. The most common natural polymer used in the bioink is collagen type 1. Natural polymers are widely used biomaterials for 3D bioprinting because of their similarities in composition to natural extracellular matrix (ECM) and their biocompatibility to the cells [38,39]. Even though natural polymers are biocompatible, they have insufficient strength to support the construct during and after printing. Hence, synthetic polymers have the advantage in providing tunable mechanical and physical properties for the bioink [36]. Another key component of the bioink is the type of cells. The cell-laden bioinks used in dental applications were mainly isolated from the human oral cavity such as dental pulp stem cells (DPSCs), periodontal ligament stem cells (PDLSCs) and stem cells from apical papilla (SCAPs). The other sources of cells used were from non-dental origin stem cells, mainly from bone marrow (BMSCs), umbilical vein (HUVECs) and amniotic fluid (AFSCs). The sources of mesenchymal stem cells (MSCs) were from humans and various animals such as porcine, rats and mice. Table 1 provides a brief summary of the cell-laden bioink with 3D bioprinting techniques for dental tissue engineering application.

This review summarizes the biomaterials and cell sources for bone, periodontal, dentin and dental pulp regeneration applications using the 3D bioprinting approach. Furthermore, the in vitro biological assessments of the 3D-bioprinted constructs towards cellular behavior activities, osteogenic/odontogenic differentiation and marker expressions are discussed. This review also highlights the in vivo studies of regenerative potential of cell-laden 3D scaffolds after implantation in various animal models.

## 2. In Vitro Assessments

In vitro biological assessments are necessary to validate 3D-bioprinted constructs based on the formulated bioink for bone, periodontal and dentin–pulp complex tissues regenerative applications. The in vitro evaluations encompass studies on cell viability, cell functionality which includes cell proliferation, cell spreading, cell migration, and cell differentiation abilities, as well as gene and protein expression after bioprinting process. Table 2 summarizes the 3D-bioprinted cell-laden that have been assessed in vitro.

### 2.1. Bone

Bone is the common type of hard tissue explored in 3D bioprinting applications in dentistry. The regeneration of craniofacial defects using conventional methods is challenging due to complex anatomical structures. The conventional methods are not predictable in reconstructing the defects and producing an accurate fit and shape [62]. Furthermore, current treatments using autogenous, allograft, xenograft and alloplast sources have their own disadvantages such as donor site morbidity, graft resorption, difficulty to conform the materials to the defects, increased risk of infection and host immune response, as well as a lack of osteogenic and osteoinductive potential [63,64,65,66]. Therefore, the ability of 3D bioprinting technology in fabricating 3D-bioprinted scaffolds with cell-laden bioinks for reconstructing defect-specific vascularized bones and facilitating the bone formation and regeneration could potentially address the current reconstructive challenges.

#### 2.1.1. Cellular Behavior Activities

High cell viability of the various 3D-bioprinted scaffolds was reported in a range of 80% to greater than 95% after printing, which showed that the printing process did not adversely affect cell viability regardless of the type of bioprinting techniques, biomaterials and cell sources. A study by Kuss et al. reported that when the stromal-vascular-fraction-derived cells, SVFC-laden hydrogel bioinks were conditioned in the (GM)/endothelial growth medium (EGM), high cell viability in both normoxic and short-term hypoxic environments was recorded at day 7. However, in long-term hypoxic conditioning of more than 14 days, the SVFC viability was significantly affected [41]. The findings by Kuss et al. were supported by other studies stating that a short-term period of hypoxia during early stages of the normal healing process of bone repair/regeneration could promote vascularization in later stages by secretion of vascular endothelial growth factor [67,68]. A novel composite bioink developed by Dubey et al. showed more ~90% viable DPSCs for up to 5 days in the culture regardless of the presence or absence of incorporation of amorphous magnesium phosphate (AMP) [43]. The DPSCs appeared elongated after 1 day within the ECM/AMP constructs [43]. Another study using DPSCs as the choice of cells in the bioink showed that human DPSCs maintained their viability above 90% and the proliferation capability for the GelMA and bone morphogenetic protein (BMP)-GelMA groups at all time points. However, there was no difference in cell behavior in the GelMA and BMP-GelMA groups, which indicates that the BMP-mimetic peptide has no influence on cell behavior [44]. Moncal et al. used the extrusion technique to fabricate a hard tissue (HT) bioink composed of collagen + chitosan + β-glycerophosphate(β-GP) + nHAp. The results showed that cell viability rBMSCs-laden HT ink was greater than 90% after bioprinting and increased to more than 95% in a week [46]. Hence, in order to maintain cell viability after printing, suitable biomaterials and cell types within the bioink should be carefully selected and the operating conditions should be optimized [69,70].

The 3D-bioprinted constructs provide a favorable microenvironment for cell proliferation. The proliferation activity of alginate + gel + nHAp/hPDLSCs bioscaffolds was higher compared to that of alginate/hPDLSCs at day 4 and day 6. The addition of nHAp in the alginate + gel scaffolds resulted in better hPDLSCs adhesion due to the rough surface of the bioscaffold [45]. In 3D-bioprinted constructs of collagen + chitosan + nHAp + β-GP treated with the growth medium, there was a significant proliferation of rBMSCs and cell migration out of the constructs between day 4 and day 7 in comparison to constructs in the osteogenic medium [46]. In another study, Moncal et al. reported that the bioink containing a platelet-derived growth factor (PDGF) showed an increase in cell proliferation ability compared to the BMP-2 group [47]. The addition of PDGF resulted in an increased proliferation of cell rate in mitogenesis [71]. Hence, the delivery of growth factors incorporated within the bioscaffolds could improve bone regeneration. The combination of GelMA/PEGDA is widely used for biomedical application and can serve as ECM mimics. By tuning the volume ratio of the two compositions, the physicochemical and biocompatibility of GelMA/PEGDA can be improved by the 3D bioprinting system. Ma et al. reported that PDLSCs encapsulated in GelMA/PEGDA had significant cell proliferation and spreading to form interconnected networks between the cells [51]. It also showed robust cytoskeletal organization in composite hydrogel with volume proportion of GelMA to PEGDA of 4:1. The cell spreading was enhanced as the volume proportion of GelMA to PEGDA increased from 1:4 to 4:1. It was shown that the cell proliferation and spreading is inhibited by more PEGDA components in the hydrogel due to its non-adhesive nature [51].

#### 2.1.2. Differentiation Activities (Alkaline Phosphatase)

Alkaline phosphatase (ALP) activity is an indicator of the differentiation ability of the cells. It is used to measure the levels of early osteogenesis. The ALP activity in normoxia and hypoxia groups in 3D bioprinted SVFC-laden constructs did not show any difference after 21 days [41]. In the ECM/AMP cell-laden bioink, ALP activity was significantly increased compared to AMP-free activity at day 14 [43]. AMP was shown to promote rapid differentiation and mineralization of pre-osteoblasts [72]. In a different study, bioscaffold alginate + gel + nHAp/hPDLSCs showed higher ALP activity after 7 and 14 days of osteogenic induction culture compared to alginate/hPDLSCs [45]. Another study by Touya et al. showed that ALP activity increased significantly on day 14 when SCAPs were cultured with osteogenic medium compared to standard culture conditions. This indicates a lack of differentiating SCAPs triggered by mineralized ink presence without osteogenic medium [50]. The effect of ECM composition on PDLSCs differentiation showed the ALP activity in GelMA/PEGDA hydrogels was higher as the volume proportion of GelMA to PEGDA increased at day 7 and day 10. This suggests the composition of hydrogel influences the ALP activity of PDLSCs [51]. In this review, for the assessment of osteogenic differentiation ability of the cell, the majority of the studies reported an increase in the ALP activity. This indicates that the 3D-bioprinted constructs retained the biological activity of the cells.

#### 2.1.3. Expression of the Markers

Short-term hypoxic environments significantly upregulated vascularization of vascular endothelial growth factor A *(VEGFA*) and (*HIF1A*) expressions in 3D-bioprinted SVFC-laden bioink in a hybrid medium osteogenic medium (OGM)/EGM. However, it did not affect the osteogenic expression of *ALP* and osteocalcin (*OCN*) [41]. The expression of osteogenic-related genes, osteopontin (*OPN*) and collagen alpha 1 (*COL1A1*), increased in ECM/AMP scaffolds compared to the ECM at 2 weeks without the use of growth factors. The *OPN* expression showed a fourfold increase in ECM/1.0AMP scaffolds at 2 and 3 weeks, which suggests that AMP could strongly stimulate the osteogenic differentiation of stem cells and osteoblasts [43,72]. Nanosilicates in the NICE bioink increased the gene expression of *COL1A21*, *SMADs 1/4/5/7* and *SOX9*, which are involved in the endochondral differentiation of hMSCs. Transforming growth factor-β (*TGF- β*) and cadherin-11, which are linked to osteoblast development and differentiation, were shown to be upregulated. The expression of osteonectin (*SPARC*), which is necessary for collagen mineralization in bone, also increased [42]. Hard tissue (HT) ink, which consists of collagen, chitosan, nHAp and β-GP, exhibited favorable *ALP* expression before mineralization. Additionally, without the presence of osteogenic medium culture, *OPN* increased during the proliferative stage and *OCN* upregulated when the cells differentiated into osteoblasts [46]. The controlled delivery group promoted all osteogenic regulator genes, *RUNX2*, *ALP*, *BMP-2* and *OCN*, due to the pDNA incorporation in the bioprinted constructs [47]. Gene-based growth factors using non-viral gene therapy have good potential for bone regeneration application [73]. Another study by Ma et al. showed that the expression of osteogenic markers *OCN* and *OPN* were significantly increased in PDLSCs-laden GelMA/PEGDA hydrogels when the volume proportion increased from 1:4 to 4:1, which can be tuned by ECM composition [51]. The BMP-GelMA group showed a significant increase in the expression of *RUNX2* after 2 weeks compared to the GelMA group, both cultured in normal growth medium. The marker expression of *COL1A1* and *OCN* increased in all groups after 4 weeks. There was no significant difference of dentin sialophosphoprotein (*DSPP*) expression level between all medium conditions [44].

### 2.2. Periodontal Tissues

Periodontium is a complex structure consisting of periodontal ligament, cementum, alveolar bone and gingiva. Periodontal ligament (PDL) is a connective tissue interface between cementum, which is a thin layer of mineralized tissue covering the roots of the teeth, and the alveolar bones. PDL is generally composed of collagen type 1. Besides functioning as a supporting structure for the teeth, PDL is also involved in repairing damaged tissues, supplying nutrients, and playing a role in homeostasis of alveolar bones [74]. Periodontitis is a chronic inflammatory disease caused by dental plaque which interacts with host immune inflammatory response alongside other genetic, environment and lifestyle risk factors. It is characterized by the progressive destruction and irreversible loss of supporting structures of the teeth such as gingiva, periodontal ligament, cementum and alveolar bone. The current challenge faced by clinicians is to reconstruct the periodontium destroyed by periodontitis, since initial non-surgical therapy does not allow regeneration of lost periodontal tissues [75]. Guided tissue regeneration treatment and bioactive molecules are mostly constrained to three-walled periodontal defects [76]. The standard periodontal regenerative technique has limitations in restoring hierarchical organization of lost tissues as well as the complete function and structural integrity of periodontium which leads to a search of alternative methods such as tissue engineering.

#### 2.2.1. Cell Behavior Activities

The cellular viability of the 3D-bioprinted constructs was reported to be around 82% after the inkjet bioprinting process [54]. Inkjet has been used in tissue engineering applications due to high cell viability and printing resolution [77]. A study by Lee et al. reported that cell viability of the collagen/hPDLSCs bioink was comparable between printing group and seeding group 1 day after printing. However, PDL cells were not well organized and showed uneven distribution in the seeding group compared to the cells in the printing group which were homogenous, well aligned and had direction. The printing group also showed more proliferation of PDLSCs on day 7 of culture [52]. This suggests that the cell printing method is more reliable than the seeding approach. The cell encapsulated printed constructs could enhance the position of cells and eliminate the possibility of poor cellular performance in the cell-seeding scaffolds [22]. In a previous study, the hPDLSCs viability decreased significantly with the increasing volume ratio of PEG to GelMA [53]. This may be attributed to the different bioactivities of PEG which can cause reduced cell growth due to non-degradable and chemically inert properties [78]. Human PDLSCs spread and elongate when the ratio of GelMA increases to a PEG volume ratio after 3 days of culture [54]. The bi-layer hGF-laden collagen/strontium-doped calcium silicate (SrCS) bioscaffold showed a higher proliferation rate compared to single layer scaffold at 3, 7 and 14 days of culture [53]. The bi-layer bioink could enhance cellular proliferation due to the release of silicon and strontium ions from the SrCS supporting base [79].

#### 2.2.2. Expression of the Markers

The gene expression of cementum protein 1 (*CEMP1*) was significantly higher in the printing groups compared to the cell seeding groups. *COL1* and *ALP* did not show any differences at day 7 of culture. The collagen/hPDLSCs-laden bioscaffold was shown to have the capacity for cementogenesis induction [52]. PDL-derived cells have shown the ability to assist in periodontal regeneration including cementum formation [80]. *CEMP1* is a cementum marker gene that can be found in PDL cells, cementoblasts and cells around the vascular networks [81,82]. Protein expressions of ALP, BSP and OCN cultured in Wharton’s jelly mesenchymal stem cells (WJMSCs) were significantly increased in bi-layer scaffolds (Col/SrCS) at days 7 and 14, which suggested better differentiation behavior [53].

### 2.3. Dentin and Pulp

Dental pulp is a highly vascularized and unmineralized tissue composed of cells, collagen fibers and proteoglycans. It is surrounded by dentin, a mineralized hard structure of the tooth which consists of hydroxyapatite as well as an organic matrix of collagenous and non-collagenous proteins. Dental pulp is an important tissue in maintaining the tooth viability, nutrition, and sensation. The existence of caries, open restoration margins, cracks or fractures creates pathways for microorganisms and toxins to enter the pulp. This leads to irritation and inflammation of the dental pulp. If the situation is left untreated, it may lead to irreversible pulp inflammation and eventually pulp necrosis. Current conventional therapy for treating irreversible inflammation and/or necrosis of dental pulp is root canal treatment which involves the complete removal of infected pulp tissues, debridement of the canal and finally filling it with an inert biomaterial. The regenerative endodontic approach is the alternative to the current treatment for regeneration of damaged or diseased pulp vitality in order to maintain the biological function of the tooth [83].

#### 2.3.1. Cellular Behavior Activities

The cell encapsulated in the hybrid hydrogels showed a more than 90% SCAPs viability after the bioprinting process for dentin/pulp regeneration. The SCAPs-laden alginate-dentin bioink showed a significantly higher cell viability compared to that of pure alginate hydrogels after 5 days in cell culture [55]. A study by Han et al. reported that fibrin-based bioink with different fibrinogen concentration ranging from 5 to 20 mg/mL showed a >90% hDPSCs viability at day 4 [56]. The hDPSCs proliferation rate decreased with the increasing fibrinogen after 16 days of culture. The higher the fibrinogen concentration, the longer the time for the cell to spread. Another study showed the calcium silicate (CS)/GelMA bioink enhanced the proliferation rate and viability of hDPSCs as the CS concentration increased after 7 days of culture [57]. The hDPSCs viability in collagen/β-TCP (CTS-20) and bone-derived decellularized ECM (dECM-20) were greater than 95% after 1 day [60]. The cell proliferation rate in dECM-20 was higher and the cytoskeleton was more spread and developed than in CTS-20. The hDPSCs proliferation was significant in the dECM-20 due to the biochemical signals of the dECM component [60]. Demineralized dentin matrix particle (DDMp)-based bioinks showed DPSCs viability of greater than 95%, regardless of the DDMp concentrations in the growth medium for 7 days, but DPSCs proliferation rate decreased as the DDMp concentration increased [58]. The viability of SCAPs-encapsulated poloxamer hydrogels was enhanced and the number of migrated cells increased after the exposure of low-electromagnetic fields (EMFs) of 5 V–1 Hz applied for 30 min per day after 3 days of culture [59]. EMF exposure has been shown to be biocompatible for SCAPs and could be used for dental tissue regeneration [84].

#### 2.3.2. Expression of the Markers

A study by Athirasala et al. reported that the alginate–dentin hydrogel bioink supplemented with 100 mg mL^−1^ of soluble dentin matrix molecules without the addition of odontogenic factors showed an increase in ALP protein expression. In the same study, gene expression showed an upregulated *ALP* and *RUNX2* in SCAPs-encapsulated alginate–dentin bioink after culturing in an odontogenic medium for 10 days [55]. A study by Han et al. found that the expression of odontogenic differentiation markers of hDPSCs such as dentin matrix acidic phosphoprotein 1 (*DMP-1*) and *DSPP* increased with fibrinogen concentration [56]. Accordingly, the outcome of hDPSCs can be regulated by controlling the fibrinogen concentration in the bioink. The expression of osteogenesis-related proteins, namely ALP, DMP-1, and OCN in CS/GelMA, showed an increase in secretion in the CS10 bioink at different time points of culture compared to other concentrations [57]. The expressions of osteogenic [*OPN*, *OCN* and biglycan (*BGN*)] and odontogenic (*DSPP* and *DMP-1*) genes increased in dECM-20 composite scaffolds under osteogenic differentiation medium at 28 days [60]. Another study by Han et al. showed that the expression levels of odontogenic markers *DSPP* and *DMP-1* increased in DDMp bioink after culturing in odontogenic differentiation medium for 15 days [58]. The combination of P407-encapsulated SCAPs with EMF treatment increased early and late gene marker, such as *ALP*, *Col1*, *DSPP* and *DMP-1*, expressions [59].

## 3. In Vivo Assessments

In this review, the in vivo applications for bone, periodontal and dentin tissue regeneration involve the implantation of 3D-bioprinted scaffolds into calvarium or dorsal subcutaneous using either extrusion or laser-assisted bioprinting approach. In situ or intra-operative bioprinting using the LAB technique has been performed to repair the defects on live subjects [85]. This approach could eliminate the issues of in vitro fabrication by offering immediate printing of bioink to the defect area. It is a highly accurate and personalized reconstruction approach bypassing the difficulties associated with the implantation of fabricated constructs. However, the LAB approach is more suitable for small defects and relatively flat bones [86]. Additionally, in this review, the studies mostly used small animal models such as mice, rats and rabbits as they were relatively easy to handle and incurred lower managing costs [87]. The animals were healthy and there were no toxicity or side effects observed during the experimental implantation period. Table 3 summarizes the in vivo assessments and findings of 3D-bioprinted bioink for dental applications.

### 3.1. Bone

A study by Kang et al. reported that 3D-bioprinted hAFSCs-HT constructs showed newly formed vascularized bone tissues throughout the implants including at the central portion compared to the other groups of non-treated and scaffold-only constructs. These groups of non-treated and scaffold-only constructs showed limited vascularization and minimal bone tissue formation at the peripheral of the implant. The study also showed mature and vascularized bone formed in immune-deficient rats after 5 months [40]. Kuss et al. reported that the capacity of the vascular network formation within 3D-bioprinted SVFC-laden constructs showed larger lumen sizes and broader vessel area distribution in hypoxic environments. The 3D-bioprinted bone constructs with SVFC in short-term hypoxic conditioning supported in vivo vascularization and rapid anastomosis, which could enhance the bone repair in the subcutaneous mice model [41]. Another study by Kérourédan et al. reported, at 2 months of implantation, that collagen-VEGF-SCAP-bioprinted HUVECs showed an increased vessel density compared to non-implanted material or defects filled with collagen. Vascularization rate and bone regeneration rate showed a significant difference compared to random seeding conditions and both disc and crossed circle patterns of HUVECs. The results indicate that in situ printing of HUVECs using the LAB technique improved vascularization with a defined configuration in mouse calvarial bone defect, which could promote bone regeneration [49]. A study by Keriquel et al. also tested different cell printing geometries which showed an impact of in situ printing on mesenchymal stromal cells with the col + nHAp arrangement on the calvarial bone defect in favor of bone regeneration in mice models [48]. A study by Touya et al. reported that mineralized ink (MI), which consists of col + TCP/SCAPs, showed no difference in bone repair between the geometrical patterns. The MI formulation using the LAB technique failed to demonstrate a complete bone repair in a calvarial defect two months after surgery [50].

### 3.2. Periodontal Tissue

Wang et al. reported that after 12 weeks of implantation on the calvarium bone of a rabbit, bi-layered hGF-Col/SrCS scaffolds showed new bones in the core of the scaffold, in contrast to single-layered SrCS scaffolds which showed new bone growth at the periphery of the scaffold. The bone volume fractions and the trabecular thickness increased in hGF-laden bioink. The results from this study showed that bi-layered hGF-Col/SrCS could enhance bone regeneration [53]. Another study by Lee et al. reported that in the printing group, PDL-like tissue was evidently seen in parallel orientation to the surface of the implant and the cells were uniformly scattered close to the calvarium bone of an athymic rat compared to the seeding groups. Periostin, vWF, HLA and CEMP1 were expressed in the cell printing groups after 6 weeks of implantation. These findings suggest that hPDLSCs-bioprinting could be a potential method for the PDL regeneration on the 3D-printed surface of titanium [52].

### 3.3. Dentin

Kim et al. reported that after 8 weeks after implantation into the subcutaneous region, the blood vessel formation of the implanted dECM-20 increased compared to other groups. This could be due to cell-laden properties and the growth factors residing in the dECM-20 bioink. The markers of osteogenic (OPN and OCN) and odontogenic (DSPP and DMP-1) differentiation also showed strong expression in the dECM-20. Based on the findings, the proposed hDPSC-laden biocomposite could serve as a potential material for dentin regeneration [60].

## 4. Challenges and Future Perspectives

The advanced technology of 3D bioprinting is a good strategy in fabricating bioscaffolds for dental applications. The selection of bioinks is crucial in determining the success of bioprinted scaffolds. The bioink should have an ideal combination of desirable physical and biological properties. The physical properties of the bioink should comprise a good viscosity for printability and reproducibility of the constructs as well as the required strength and elasticity for mimicking the mechanical properties of native tissues. These will ensure the printed structures have favorable biological properties that allow adequate cell survival, cell growth and cell differentiation in suitable environments. It is evident from this review that cell-laden bioink produces 3D-bioprinted structures with desirable biological properties. High cell viability of the constructs was maintained throughout the printing and post-printing processes regardless of the bioink composition (biomaterials and cells) and the 3D-bioprinting techniques. The biocompatible bioink is able to facilitate the printing process and act as a cell carrier allowing the growth of the cells [88]. Maintenance of high cell viability is essential to ensure effective migration, spreading and proliferation of the cells as well as differentiation ability in order to achieve geometrical, mechanical and biological similarity to native tissues [70,89]. However, the availability of wide heterogeneity of cell-laden bioink with similar good results imposes another set of challenges to the end users in deciding which bioink has superior outcomes in terms of restoring the function of lost or injured tissues. The difficulties in choosing the most suitable bioink may hinder clinical translation since various types of tissues require different properties of biomaterials and sources of cells for dental applications. It is our hope that future research breakthroughs in bioink would bring the much-needed standardization and close similarity to normal tissues.

The studies in this review mainly explored the in vitro assessments of the 3D constructs which could not yet prove their effectiveness in vivo. Only a few studies tried to address more complicated assessments such as angiogenesis and vasculogenesis. Vascularization plays an important role in bone formation and determining the success of the whole regeneration process. Even though the current state of 3D bioprinting allows the achievement of sizable living tissue constructs that mature into vascularized tissues in vivo, it is still a major challenge to achieve reproducible complex large architectures that are well vascularized for clinical usage. In large constructs, the printing time becomes longer, and this process decreases cell viability resulting in low vascular supply to the region [1]. Future research on vascularization of the scaffolds in 3D bioprinting could be further developed to avoid necrotic failure of the implantation. 

For periodontal regeneration, there are challenges that need to be addressed in future studies such as designing scaffolds that mimic the compositions of the extracellular matrix of periodontal tissues, have the ability to reconstruct the complex hierarchical architecture of periodontium, and have the capability to regenerate large periodontal defects. The fabrication of multiphasic constructs of different region-specific patterns and pore sizes with spatiotemporal delivery of bioactive cues is designed for integrated multi-layer periodontal tissue regeneration [90]. This can be a valuable strategy in designing customized 3D constructs by combining biomaterials and the desired cells to better match the architecture of the native periodontal tissues in order to enhance both soft (periodontal ligament) and hard tissue (alveolar bone and cementum) regeneration [53]. The microextrusion-based 3D bioprinting is suitable for rapid fabrication of large and complex cell-laden constructs which consist of multiple biomaterials [91]. To the best of the authors’ knowledge, the present studies are still at the preliminary stages, and they report no success in regenerating multi-layer cementum–periodontal ligament–alveolar bone components simultaneously with proper orientation and integration to the surrounding structures using the 3D bioprinting approach. 

In addition, there has not been any major success in pulp regeneration via 3D bioprinting due to the challenges of cell survivability in root canals, revascularization and reinnervation of pulp tissues. Prevascularized pulp tissue-like fabrication combining multiple cell types, microvessels and extracellular matrix can be an important strategy to generate preformed microvascular networks in the tissue constructs prior to implantation. The vessel-like structures can be quickly anastomosed with the host blood vessels post-implantation and further develop adequate vascularization and perfusion to ensure the survival of the engineered tissue constructs [92]. However, a limited amount of the available literature reports the vasculogenesis (new hollow capillary) formation inside the engineered tissue in in vitro studies. Nevertheless, this evidence has not been proven in in vivo models to determine the success of dental pulp tissue regeneration and the restoration of biological function of the tooth.

Even though the novel biomaterials and stem-cell-based approach using the 3D bioprinting method have shown promising results in both in vitro and in vivo assessments, unfortunately, the authors believe the technology is not yet ready to become a clinical reality. Further studies are needed for in vivo application, especially in large animal models such as dogs, sheep or monkeys to gain a better understanding of the bioprinted scaffolds prospects for clinically relevant size and architecture [93]. Despite the fact that it has managed to provide a proof-of-concept validation use for dental tissue regeneration, results in small animals should be interpreted with caution when the findings are used for human clinical applications [94,95]. The implantation of the 3D bioprinted scaffolds would be ideal if the research model could be performed under a complex oral environment reflecting true oral cavity condition. 

However, it must be noted that there was significant progress, albeit at preliminary stages, in pre-clinical research for dental applications. The general process for 3D bioprinting of dental tissues involves (i) the 3D modeling of the images based on digital scanning of the defect or area of interest, (ii) the isolation and differentiation of stem cells into dental tissue-specific cells, (iii) preparation of the bioink and loading into the printer, (iv) bioprinting of the desired structure and (v) architectural reconfiguration or chemical functionalization prior to implantation. The translation of bioprinted constructs to clinical practice is yet to be explored as the technical, ethical and legal issues need to be addressed for efficient and safe applications in humans. The issue involving labor-intensive techniques in obtaining potential donor sites and in isolating and culturing cells before implantation is a significant challenge as the potential of cells to expand and to survive during implantation is crucial for clinical translation [96]. There are also concerning issues of using living stem cells in 3D bioprinting which include the undesired lineage of the cells, the unpredictable behavior, and the long-term effects such as immunological responses and tumor formation [97,98]. 

Apart from that, the cost of the 3D bioprinter is another factor that needs to be considered for mainstream clinical applications. High-end commercial 3D bioprinters, which are more popular and preferable for usage in research environments in universities and hospitals, are very much costly compared to the commercially available low-end 3D bioprinters. The major advantages of these high-end 3D bioprinters are related to their capabilities of handling multiple biomaterials with precise positioning and dispensing of different ranges of cells. Additionally, these costly 3D bioprinters have the advantages of multiaxial printing with different degrees of motion, rapid fabrication for high-throughput production, fine printing resolution with higher positioning accuracy, repeatability and reproducibility of the process, and the ability to maintain sterilized printing environment of the bioprinted constructs when compared to low-cost bioprinters [99]. 

However, as the demand for 3D bioprinting technology keeps on increasing, it would naturally lead to more commercial manufacturers of 3D bioprinters (extrusion, inkjet and laser-based techniques) entering the supply side with competitive pricing strategies, comparable capabilities, and good specifications in order to capture some of the market. This would eventually make the 3D bioprinters more accessible and much more cost-efficient for everyone in the near future. 

Finally, on the issue of regulatory control in using human cells or tissues, this requires special attention in order to prevent significant harm prior to the adoption of 3D bioprinting technology in patients. The authors believe that for the regulatory control to be effective, all stakeholders including the lawmakers, legal experts, scientists, clinicians, industrial partners, and society at large must be able to have transparent and inclusive discussions with the common aim of advancing safe and effective health care treatment.

## 5. Conclusions

Three-dimensional bioprinted constructs have shown favorable in vitro results in maintaining cell viability and in promoting growth, proliferation, and differentiation ability of the cells, as well as in vivo after implantation in small animal models. Despite the promising progress towards the implementation of 3D bioprinting in dentistry, more pre-clinical and clinical studies are required in order to address the technical, ethical and legal issues. The potential for clinical translation in bone, periodontal, dentin and pulp tissues regeneration is very encouraging.

## Figures and Tables

**Table 1:** Three-dimensional bioprinting techniques and strategy for dental applications.

Tissue Type	Bioprinting Technique	3D Bioprinter	Cell-Laden Bioink	Cell Types	Study Design	Author
Bone	Extrusion	Integrated tissue–organ printing system	Gelatin + fibrinogen + HA + glycerol	hAFSCs	In vitro and in vivo	Kang et al., 2016 [40]
	Extrusion	3D Bioplotter (EnvisionTEC GmbH, Gladbeck, Germany)	MeHA + GelMA + HA	Porcine stromal vascular fraction from adipose tissue	In vitro and in vivo	Kuss et al., 2017 [41]
	Extrusion	Modified ANET A8 3D printer, Shenzhen, China	GelMA + kCA + nSi(NICE bioink)	Human primary bone-marrow-derived MSCs	In vitro	Chimene et al., 2020 [42]
	Extrusion	3DDiscovery, regenHU, Villaz-St-Pierre, Switzerland	ECM + AMP	DPSCs	In vitro	Dubey et al., 2020 [43]
	Extrusion	Integrated tissue–organ printing system	Gelatin + GelMA + HA + glycerol	DPSCs	In vitro	Park et al., 2020 [44]
	Extrusion	3D Bioplotter (EnvisionTEC GmbH, Gladbeck, Germany)	Alginate + gelatin + nHAp	hPDLSCs	In vitro	Tian et al., 2021 [45]
	Extrusion	In-house developed MultiArm Bioprinter, Iowa City, IA, USA	Collagen + chitosan + β-glycerophosphate + nHAp	Rat BMSCs	In vitro	Moncal et al., 2021 [46]
	Extrusion	In-house developed MultiArm Bioprinter, Iowa City, IA, USA	Collagen + chitosan + β-glycerophosphate + nHAp	Rat BMSCs	In vitro	Moncal et al., 2022 [47]
	LAB	LAB workstation (U1026, Inserm, Bordeaux, France)	Collagen type 1 + nHAp	Mouse bone marrow stromal precursor D1 cell line	In vitro and in vivo	Keriquel et al., 2017 [48]
	LAB	LAB workstation (U1026, Inserm, Bordeaux, France)	Collagen type 1	SCAPs and HUVECs	In vivo	Kérourédan et al., 2019 [49]
	LAB	LAB workstation (U1026, Inserm, Bordeaux, France)	Collagen type 1 + TCP (BioRoot RCS^®^, Septodont, France)	SCAPs	In vitro and in vivo	Touya et al., 2022 [50]
	Inkjet	Customer-designed pressure-assisted valve-based bioprinting system	GelMA + PEGDA	Rat PDLSCs	In vitro	Ma et al., 2017 [51]
Periodontal	Extrusion	3DX Printer, T and R Biofab Co., Ltd., Siheung, Korea	Collagen	hPDLSCs	In vitro and in vivo	Lee et al., 2021 [52]
	Extrusion	BioScaffolder 3.1, GeSiM, Groβerkmannsdorf, Germany	Collagen	Human gingiva fibroblasts	In vitro and in vivo	Wang et al., 2021 [53]
	Inkjet	Customer-designed pressure-assisted valve-based bioprinting system	GelMA + PEGDA	hPDLSCs	In vitro	Ma et al., 2015 [54]
Dentin & Pulp	Extrusion	Hyrel 3D, Norcross, GA, USA	Alginate + dentin matrix	SCAPs	In vitro	Athirasala et al., 2018 [55]
	Extrusion	Integrated tissue–organ printing system	Gelatin + fibrinogen + HA + glycerol	DPSCs	In vitro	Han et al., 2019 [56]
Dentin	Extrusion	BioX, CELLINK, Gothenburg, Sweden	Calcium silicate + GelMA	DPSCs	In vitro	Lin et al., 2021 [57]
	Extrusion	Homemade 3D bioprinter, Ulsan, Korea	Demineralized dentin matrix particles + fibrinogen + gelatin	DPSCs	In vitro	Han et al., 2021 [58]
	Extrusion	CELLINK BIO-X 3D printer, Gothenburg, Sweden	Poloxamer-407	SCAPs	In vitro	Dutta et al., 2021 [59]
	Extrusion	DTR3-2210 T-SG; DASA Robot, Bucheon, Korea	Collagen type 1 or dECMs + β-TCP	DPSCs	In vitro and in vivo	Kim et al., 2022 [60]
Pulp	Inkjet	Hand-held bioprinter (DropGun, BlackDrop Biodrucker GmbH, Aachen, Germany)	Collagen type 1 + agarose	DPSCs & HUVECs	In vitro	Duarte Campos et al., 2020 [61]

AMP, amorphous magnesium phosphates; BMSCs, bone marrow mesenchymal stem cells; dECM, decellularized extracellular matrix; DPSCs, human dental pulp stem cells; ECM, extracellular matrix; GelMA, gelatin methacryloyl; HA, hyaluronic acid; hAFSCs, human-amniotic-fluid-derived stem cells; hPDLSCs, human periodontal ligament stem cells; HUVECs, human umbilical vein endothelial cells; kCA, kappa-carrageenan; LAB, laser-assisted bioprinting; MeHA, methacrylated hyaluronic acid; MSCs, mesenchymal stem cells; nHAp, nano hydroxyapatite; nSi, nanosilicates; PEGDA, poly(ethylene glycol) dimethacrylate; Poloxamer-407, synthetic copolymer of poly(ethylene glycol) and poly(propylene glycol); SCAPs, human stem cells from apical papilla; TCP, tricalcium phosphate.

**Table 2:** In vitro biological assessments on 3D-bioprinted constructs for dental applications.

Tissue Type	Bioprinting Technique	Bioink	Assessments	Outcomes	Cell Densities	Cell Viability	Author
Bone	Extrusion	Gelatin + fibrinogen + HA + glycerol	1. Cell viability 2. Cell proliferation 3. Osteogenic differentiation	1. Printing process did not adversely affect cell viability at day 1 of culture 2. Cell proliferation increased over 15-day period 3. Calcium deposition in the hAFSCs-laden hydrogel in 3D bone structures	5 × 10^6^ cells/mL	91 ± 2%	Kang et al., 2016 [40]
	Extrusion	MeHA + GelMA + HA	1. Cell viability 2. Alkaline phosphatase activity 3. Gene expression	1. SVFC in bioprinted constructs showed high cell viability in both normoxic and hypoxic environments at day 7; however, long-term hypoxia (more than 14 days) impaired cell viability and vascularization 2. No significant difference in ALP activity between normoxia and hypoxia groups (after 21 days) in 3D bioprinted bone constructs using SVFC-laden hydrogels and PCL/HAp 3. Short-term hypoxia promoted vascularization of SVFC by significantly upregulating *VEGFA* and *HIF1A* expression in SVFC-laden hydrogels culture in GM/EGM	4 × 10^6^ cells/mL	-	Kuss et al., 2017 [41]
	Extrusion	GelMA + kCA + nSi (NICE bioink)	1. Cell-assisted matrix remodeling (histological) 2. Calcium content 3. Gene expression	1. Cells deposit cartilage/osteoid-like matrix of glycosaminoglycans, proteoglycans and collagen followed by mineralization of the surrounding matrix 2. Calcium content increased steadily from day 0 to day 60 3. Upregulated gene expression of *SMADs* 1/4/5/7, *SOX9*, *TGF-β*, osteonectin (*SPARC*), cadherin-11	-	-	Chimene et al., 2020 [42]
	Extrusion	ECM + AMP	1. Cell viability 2. Osteogenic differentiation 3. Gene expression	1. Cell-laden bioink with and without AMP showed viable cells ~90% up to day 5 2. Cell-laden bioprinted constructs with AMP showed high level of ALP activity 3. ECM/AMP bioink increased *OPN* and *COL1A1* osteogenic gene expression at day 14	1 × 10^6^ cells/mL	>90%	Dubey et al., 2020 [43]
	Extrusion	Gelatin + GelMA + HA + glycerol	1. Cell viability 2. Cell proliferation 3. Osteogenic differentiation 4. Gene expression	1. hDPSCs viability was >90% for bioprinted GelMA and BMP-GelMA constructs at all time points 2. hDPSCs maintained the proliferation capability in both constructs 3. BMP mimicking peptide can promote osteogenic expression 4. Increase in the expression of *RUNX2* at 2 weeks in BMP-GelMA compared to GelMA group, both cultured in normal growth medium. *COL1A1* and *OCN* expression increased in all groups after 4 weeks. No significant difference of expression level of *DSPP* in all medium conditions	-	>90%	Park et al., 2020 [44]
	Extrusion	Alginate + gelatin + nHAp	1. Cell viability 2. Cell adhesion 3. Cell proliferation 4. ALP activity	1. hPDLSCs were viable in alginate + gel + nHAp and alginate only bioscaffolds 2. Cell adhesion of alginate + gel + nHAp bioscaffold was better than that of alginate only 3. Cell proliferation activity rate of alginate + gel + nHAp bioscaffold was higher than that of alginate only at days 2, 4 and 6 4. ALP activity of alginate + gel + nHAp was higher than that of alginate only bioscaffold after 14 days	-	-	Tian et al., 2021 [45]
	Extrusion	Collagen + chitosan + β-glycerophosphate + nHAp	1. Cell viability 2. Cell proliferation 3. Gene expression	1. Cell viability after printing increased to >95% in a week 2. rBMSCs significantly proliferated between day 4 and day 7 3. *ALP*, *OPN* and *OCN* were upregulated and showed favorable osteogenic properties	5 × 10^6^ cells/mL	>95%	Moncal et al., 2021 [46]
	Extrusion	Collagen + chitosan + β-glycerophosphate + nHAp	1. Cell viability 2. Cell proliferation 3. Calcium deposition 4. Protein expression 5. Gene expression	1. Cell viability after printing increased to >95% in a week 2. Bioink + PDGF groups resulted in increase in cell proliferation rate compared to the BMP-2 group 3. Bioink + pPDGF-B + CS-NPs (pBMP-2) had a significant increase in calcium ion deposition in week 3 compared to other groups 4. Bioink + CS-NPs (pBMP-2) group had the highest BMP-2 production on day 4 compared to other groups 5. All osteogenic regulator genes, *RUNX2*, *ALP*, *BMP-2* and *OCN* indicated that Bioink + pPDGF-B + CS-NPs(pBMP-2) promoted the most accelerated osteogenic differentiation	8 × 10^5^ cells/mL	>95%	Moncal et al., 2022 [47]
	LAB	Collagen type 1 + nHAp	1. Cell proliferation 2. Metabolic activity	1. Cells proliferated and fill the voids at day 2 and day 4 2. Cells showed an increase in metabolic activity from day 1 to day 8	120 × 10^6^ cells/mL	-	Keriquel et al., 2017 [48]
	LAB	Collagen type 1 + TCP (BioRoot RCS^®^, Septodont, France)	1. ALP activity 2. Osteogenic differentiation 3. Cell migration	1. Cells in osteogenic medium expressed higher ALP activity compared to other conditions at day 14 2. The use of mineralized ink (MI) was not able to meet the level of osteogenic differentiation with a dedicated medium 3. Cell migration speed was found to be enhanced by the presence of MI	2 × 10^3^ cells/mL	-	Touya et al., 2022 [50]
	Inkjet	GelMA + PEGDA	1. Cell viability 2. Cell proliferation 3. Cell spreading 4. ALP activity 5. Gene expression	1. PDLSCs viability was ~90% in the composite hydrogels with GelMA to PEGDA volume proportion of 2:3 to 4:1 2. GelMA/PEGDA proportion of 4:1 hydrogel showed significant PDLSCs proliferation after 7 days 3. PDLSCs spreading was enhanced as the volume proportion of GelMA to PEGDA increased 4. ALP activity of PDLSCs increased as the volume proportion of GelMA to PEGDA increased at day 7 and day 10 5. *OCN* and *OPN* expression of PDLSCs were increased when the volume proportion of GelMA/PEGDA increased from 1:4 to 4:1	1 × 10^6^ cells/mL	~90%	Ma et al., 2017 [51]
Periodontal	Extrusion	Collagen	1. Cell viability 2. Cell proliferation	1. Cell viability was lower in cell printing group compared to cell seeding group on day 1 with no significant difference 2. Cell proliferation in cell printing group showed good extent on day 7	1 × 10^7^ cells/mL	-	Lee et al., 2021 [52]
	Extrusion	Collagen	1. Cell viability 2. Cell proliferation 3. Quantification of growth factors 4. Protein expression	1. No dead cells in Col-based bioink at week 0, 1, 2, 4, 6 and 8 2. Proliferation levels were higher in bi-layer scaffold (Col/SrCS) compared to one-layer Col bioink at days 3, 7 and 14 3. Bi-layer group had higher secretions of FGF-2, BMP-2 and VEGF from human gingival fibroblasts at all time points 4. Increased secretion of osteogenic-related proteins ALP, BSP and OC from the bi-layer scaffold (Col/SrCS) at days 7 and 14	5 × 10^5^ cells/mL	-	Wang et al., 2021 [53]
	Inkjet	GelMA + PEGDA	1. Cell viability 2. Cell spreading 3. Cell proliferation	1. PDLSCs viability was 82.4 ± 4.7% after 72 h for a pressure range of 40–60 kPa 2. Spreading area of PDLSCs reduced dramatically with a decrease in GelMA and increase in PEG volume proportion 3. Viable cells decreased with decreasing proportion of GelMA on day 3 and day 5	1 × 10^6^ cells/mL	82.4 ± 4.7%	Ma et al., 2015 [54]
Dentin & Pulp	Extrusion	Alginate + dentin matrix	1. Cell viability 2. Protein expression 3. Gene expression	1. Cells encapsulated in Alg-Dent hydrogels had higher cell viability >90% after 5 days 2. Increased expression of ALP at the protein levels in cell-laden bioink 3. Increased in *ALP* and *RUNX2* gene expression in cell-laden bioink after 10 days	0.8 × 10^6^ cells/mL	>90%	Athirasala et al., 2018 [55]
	Extrusion	Gelatin + fibrinogen + HA + glycerol	1. Cell viability 2. Cell proliferation 3. Gene expression	1. Viability of hDPSCs was >90% in all groups at day 4 2. hDPSCs proliferation rate decreased with increasing fibrinogen concentration 3. Expression of *DMP-1* and *DSPP* increased with fibrinogen concentration	3 × 10^6^ cells/mL	>90%	Han et al., 2019 [56]
Dentin	Extrusion	Calcium silicate + GelMA	1. Cell viability 2. Cell proliferation 3. Calcium deposition 4. Protein expression	1. hDPSCs viability increased when CS concentration increased in CS/GelMA bioink 2. CS/GelMA bioink enhanced the proliferation rate of hDPSCs on day 7 as the concentration of CS increased 3. Calcium deposition increased in CS10 group at day 7 and day 14 4. The expressions of ALP, DMP-1 and OC were enhanced from the release of silicon ions in CS/GelMA bioink	5 × 10^6^ cells/mL	-	Lin et al., 2021 [57]
	Extrusion	Demineralized dentin matrix particles (DDMp) + fibrinogen + gelatin	1. Cell viability 2. Cell proliferation 3. Osteogenic differentiation 4. Gene expression	1. Viability of DPSCs > 95% in all concentrations of DDMp bioinks and fibrinogen-gelatin mixture at day 7 2. DPSCs proliferation rate decreased as the DDMp concentration increased at day 7 3. Higher mineralization in DDMp bioink group compared to fibrogen–gelatin mixture after culturing with differentiation medium for 15 days 4. Expression levels of *DSPP* and *DMP-1* were higher in DDMp bioink	3 × 10^6^ cells/mL	>95%	Han et al., 2021 [58]
	Extrusion	Poloxamer-407	1. Cell viability 2. Cell morphology 3. Cell migration 4. Gene expression	1. SCAPs viability increased in 5 V-1 Hz (0.62 mT) EMF exposure after 3 days of culture 2. The entire 3D matrix was covered by cells in 5 V EMF-treated groups after 3 days of culture 3. The number of migrated cells increased in EMF-treated and PAI-1 + EMF-treated samples 4. Higher expression of *ALP*, *DSPP*, *DMP-1* and *Col-1* in 5 V EMF treatment	2.5 × 10^4^ cells/mL	-	Dutta et al., 2021 [59]
	Extrusion	Collagen type 1 or dECMs + β-TCP	1. Cell viability 2. Cell proliferation 3. Gene expression	1. hDPSCs viability in collagen/β-TCP (CTS-20) and dECM/β-TCP (dECM-20) were approximately 97% after 1 day 2. Cell proliferation in dECM-20 bioink was higher than CTS-20 3. Significant increase in osteogenic gene expression of *OPN*, *OCN*, *BGN* and odontogenic gene expression of *DSPP* and *DMP-1* in dECM-20	1 × 10^7^ cells/mL	>95%	Kim et al., 2022 [60]
Pulp	Inkjet	Collagen type 1 + agarose	1. Vasculogenesis	1. Vascular tube formation in all tested hydrogels	3 × 10^6^ cells/mL	-	Duarte Campos et al., 2020 [61]

3D, three-dimensional; ALP, alkaline phosphatase; AMP, amorphous magnesium phosphates; BGN, biglycan; BMP, bone morphogenetic protein; BMSCs, bone marrow mesenchymal stem cells; BSP, bone sialoprotein; Col, collagen; COL1A1, collagen alpha 1; CS-NPs(pBMP-2), chitosan-nanoparticle encapsulating DNA encoded with bone morphogenetic protein-2; dECM, decellularized extracellular matrix; DMP-1, dentin matrix acid phosphoprotein; DPSCs, human dental pulp stem cells; DSPP, dentin sialophosphoprotein; ECM, extracellular matrix; EGM, endothelial medium; EMF, electromagnetic fields; FGF, fibroblast growth factor; GelMA, gelatin methacryloyl; GM, growth medium; HA, hyaluronic acid; hADSCs, human-adipose-tissue-derived mesenchymal stem cells; hAFSCs, human-amniotic-fluid-derived stem cells; HAp, hydroxyapatite; HIF1A, hypoxia inducible factor 1 subunit alpha; hPDLSCs, human periodontal ligament stem cells; HUVECs, human umbilical vein endothelial cells; Hz, hertz; kCA, kappa-carrageenan; LAB, laser-assisted bioprinting; MeHA, methacrylated hyaluronic acid; MSCs, mesenchymal stem cells; nHAp, nano hydroxyapatite; nSi, nanosilicates; OCN, osteocalcin; OPN, osteopontin; PAI-1, plasminogen activator inhibitor-1; PCL, polycaprolactone; PDGF, platelet-derived growth factor; PEGDA, poly(ethylene glycol) dimethacrylate; Poloxamer-407, synthetic copolymer of poly(ethylene glycol) and poly(propylene glycol); pPDGF-B, platelet-derived growth factor-B encoded plasmid-DNA; RUNX2, runt-related transcription factor 2; SCAPs, human stem cells from apical papilla; SrCS, strontium-doped calcium silicate; SVFC, stromal vascular fraction derived cells; TCP, tricalcium phosphate; TGF-β, transforming growth factor-β; V, volt; VEGFA, vascular endothelial growth factor A.

**Table 3:** In vivo assessments on 3D-bioprinted constructs for dental applications.

Tissue Type	Bioprinting Technique	Bioink	Animal Model	Defect Area	In Vivo Testing	Outcomes	Author
Bone	Extrusion	Gelatin + fibrinogen + HA + glycerol	Sprague Dawley rats 250–300 g	Calvarium 8 mm diameter, 1.2 mm depth	1. Histology 2. Immuno-histology	1. Bioprinted materials showed newly vascularized bone tissues with no necrosis at implanted sites 2. Large blood vessel formation within newly formed bone tissues	Kang et al., 2016 [40]
	Extrusion	MeHA + GelMA + HA	Female athymic nude mice 8 weeks old	Dorsal sub-cutaneous	1. Histology 2. Immuno-histology 3. Microvessel density and area distribution	1. Dense populated cells with obvious microvascularity throughout the bioprinted constructs 2. Integration of formed lumens with existing host vasculature 3. Lumen sizes were larger, and broader vessel area distribution in constructs conditioned with hypoxic environment compared to normoxia group	Kuss et al., 2017 [41]
	LAB	Collagen type 1 + nHAp	Female Balb/c mice 12 weeks old 19–20 g	Calvarium 3.3 mm diameter	1. Micro-CT 2. Histology	1. Increase in BV/TV at 2 months after printing in nHAp-collagen-D1 cells with disk geometry 2. Substantial and well-distributed new bone formation throughout the defect at 1 month and formation of mature bone at the center of the defect at 2 months in nHAp-collagen-D1 cells with disk geometry	Keriquel et al., 2017 [48]
	LAB	Collagen type 1	Female NSG mice 10 weeks old 25–26 g	Calvarium 3.3 mm diameter	1. Fluorescence imaging 2. Micro-CT 3. Histology	1. Vascular network were well interconnected in printed pattern when compared to randomly seeded cells which had weak network organization into the defect 2. Increased percentage bone formation (BV/TV) in printed HUVECs in calvarial defects at 2 months 3. Printed HUVECs increased the vessel density in bone defects at 2 months	Kérourédan et al., 2019 [49]
	LAB	Collagen type 1 + TCP (BioRoot RCS^®^, Septodont, France)	Female NSG mice 8 weeks old	Calvarium 3.3 mm diameter	1. Micro-CT 2. Histology	1. Mineralized Ink (MI) was found not to be effective in improving bone repair and there was no difference between the two patterns after 2 months 2. No difference in vessel density between defects filled with MI, control and pipette deposit	Touya et al., 2022 [50]
Periodontal	Extrusion	Collagen	Male athymic rats 9 weeks old	Calvarium 8 mm diameter, 1.2 mm depth	1. Histology 2. Immunohisto-chemistry	1. Fibrous connective tissue was apparent in the cell printing group which was not observed in the seeding group. Periodontal-like tissue was oriented parallel to the porous titanium implant surface in the cell printing group 2. HLA, periostin, vWF and CEMP1 were expressed in the connective tissues produced in the cell printing groups	Lee et al., 2021 [52]
	Extrusion	Collagen	Female New Zealand white rabbits 2 kg	Calvarium 7 mm diameter, 8 mm depth	1. Micro-CT 2. Histology	1. hGF-laden bi-layered scaffolds had higher Tb.Th and BV/TV ratio after 12 weeks of implantation 2. hGF-laden bi-layered scaffolds were wrapped by new bone tissues compared to SrCS scaffold which had new bone growth at the periphery of the scaffold	Wang et al., 2021 [53]
Dentin	Extrusion	Collagen type 1 or dECMs + β-TCP	Athymic nude mice	Dorsal sub-cutaneous	1. Histology 2. Immuno-histochemistry	1. Increase in blood vessel formation in the implanted dECM-20 scaffold 2. Strong signal of OPN and OCN in dECM-20 DSPP and DMP-1 were strongly expressed in the dECM-20 at 8 weeks	Kim et al., 2022 [60]

BV/TV, bone volume/total volume; CEMP1, cementum protein 1; dECM, decellularized extracellular matrix; DMP-1, dentin matrix acid phosphoprotein; DSPP, dentin sialophosphoprotein; GelMA, gelatin methacryloyl; HA, hyaluronic acid; hGF, human gingival fibroblast; HUVECs, human umbilical vein endothelial cells; LAB, laser-assisted bioprinting; MeHA, methacrylated hyaluronic acid; Micro-CT, microcomputed tomography; nHAp, nano hydroxyapatite; OCN, osteocalcin; OPN, osteopontin; PEGDA, poly(ethylene glycol) dimethacrylate; SrCS, strontium-doped calcium silicate; Tb.Th, trabecular thickness; TCP, tricalcium phosphate.

## Data Availability

Not applicable.
